# Immune cells and preterm labour: do invariant NKT cells hold the key?

**DOI:** 10.1093/molehr/gav002

**Published:** 2015-01-14

**Authors:** S.F. Rinaldi, A.G. Rossi, P.T.K. Saunders, J.E. Norman

**Affiliations:** 1 MRC Centre for ReproductiveHealth and Tommy's Centre for Maternal and Fetal Health, University of Edinburgh, Queen's Medical Research Institute, Edinburgh, UK; 2 MRC Centrefor Inflammation Research, University of Edinburgh, Queen's Medical Research Institute, Edinburgh, UK

Whilst there is now considerable evidence that labour, both at term and preterm, is an inflammatory event associated with an immune cell influx into the utero-placental tissues (Thomson *et al.*, 1999; Young *et al.*, 2002; Osman *et al.*, 2003; Gomez-Lopez *et al.*, 2010; Rinaldi *et al.*, 2011; Hamilton *et al.*, 2012), the precise role that these immune cells play in the initiation of labour, either in the presence of an intrauterine infection preterm, or physiologically at term, is unclear. Previous work has shown that the infiltrating leukocytes are a major source of pro-inflammatory mediators, including interleukin (IL)-1β, IL-8, IL-6, tumour necrosis factor (TNF)-α and MMP-9 (Roh *et al.*, 2000; Helmig *et al.*, 2002; Young *et al.*, 2002), suggesting that these immune cells are likely to contribute to the inflammatory response surrounding parturition. However, whether the infiltration of leukocytes is a key event in triggering the onset of parturition or merely a consequence of the heightened inflammatory signalling remains to be elucidated.

*In vivo* animal models have been an invaluable tool to study the role of immune cells in parturition onset, where in recent years several studies have utilized either antibody-mediated immune cell depletion or genetically modified mice to investigate the function of specific immune cell populations in normal and adverse pregnancy outcomes.

In this issue of *Molecular Human Reproduction*, Li *et al*. build upon their previous findings by identifying the molecular mechanisms underlying the role of activation of invariant natural killer T (iNKT) cells in inflammation-induced preterm birth in a mouse model (Li *et al.*, 2012, 2015).

iNKT cells are a subset of T cells which express both NK cell receptors and a rearranged T cell receptor (TCR) which has a semi-invariant TCRα chain (in mice this is a Vα14-Jα18 rearrangement, and in humans a Vα24-Jα18 rearrangement), that pairs with a restricted set of TCRβ chains (Van Kaer *et al.*, 2013). This TCR rearrangement restricts iNKT cells to recognize self and foreign lipids presented by the MHC class 1-related protein CD1d (Brennan *et al.*, 2013). Upon activation, iNKT cells rapidly secrete cytokines, such as interferon (IFN)-γ and IL-4, and these cytokines can activate other immune cells, such as NK cells, dendritic cells, macrophages, neutrophils, B cells and T cells, and can therefore participate in a wide range of immune responses (Brennan *et al.*, 2013). Due to their ability to produce a wide variety of cytokines and interact with other cells of the immune system, iNKT cells are often considered as a ‘bridge’ between the innate and adaptive immune systems (Boyson *et al.*, 2008).

Several studies have now reported the presence of iNKT cells in the pregnant human and mouse decidua (Ito *et al.*, 2000; Tsuda *et al.*, 2001; Boyson *et al.*, 2002). iNKT cell stimulation with its specific ligand α-galactosylceramide (α-GalCer) results in both early pregnancy loss and preterm birth in mouse models (Ito *et al.*, 2000; Boyson *et al.*, 2006). Additionally, Jα18 KO mice, which have no iNKT cells, have a reduced rate of lipopolysaccharide (LPS)-induced early pregnancy loss and LPS-induced preterm birth (Li *et al.*, 2012, 2013). In this issue, Li *et al.* report that adoptive transfer of decidual iNKT cells to Jα18 KO mice promotes LPS-induced preterm birth and, by using various neutralizing antibodies, demonstrate that decidual iNKT cell activation is regulated by TLR-4-mediated signalling pathways, IL-12 and IL-18 secretion, and endogenous glycolipid antigens presented by CD1d.

In women, neutrophils are proposed to play a role in cervical ripening (Bokström *et al.*, 1997) and have been shown to infiltrate into the myometrium and cervix in association with term labour (Thomson *et al.*, 1999; Osman *et al.*, 2003). Furthermore, neutrophil infiltration has been specifically associated with infection-induced preterm labour. Hamilton *et al.* recently reported increased decidual neutrophil infiltration in women with infection-associated preterm labour, compared with women in either idiopathic or normal term labour (Hamilton *et al.*, 2012). A similar neutrophil influx has been reported in mouse models of inflammation-induced preterm labour (Shynlova *et al.*, 2013; Rinaldi *et al.*, 2014). However, in these models, neutrophil depletion in mice has been reported to have no effect on the timing of delivery either in normal term labour (Timmons and Mahendroo, 2006) or in models of CpG-oligodeoxynucleotide (CpG ODN; a TLR-9 agonist)-induced (Thaxton *et al.*, 2009; Sun *et al.*, 2013) and LPS-induced preterm birth (Rinaldi *et al.*, 2014), suggesting that neutrophil infiltration is not required for the onset of preterm labour.

The role of mast cells in the onset of parturition has also been investigated (Menzies *et al.*, 2011). Mast cells have been identified in the pregnant human and mouse uterus and cervix (Thomson *et al.*, 1999; Naik *et al.*, 2004; Garfield *et al.*, 2006; Menzies *et al.*, 2012); and there is evidence indicating that degranulation of mast cells can induce myometrial contractility *in vitro* (Garfield *et al.*, 2000; Bytautiene *et al.*, 2004, 2008). However, mast-cell-deficient Kit^W-sh^ mice had no defects in the timing of normal term labour (Menzies *et al.*, 2012), suggesting that mast cells are not essential to the initiation of parturition in mice. Whether mast cells may be involved in the onset of inflammation-induced preterm birth has yet to be determined.

Conversely, macrophages appear to play a more crucial role in the onset of parturition. Macrophages account for around 20% of the decidual leukocyte population (Erlebacher, 2013) and there is an influx of macrophages into the myometrium, fetal membranes, decidua, placenta and cervix during spontaneous term labour (Thomson *et al.*, 1999; Osman *et al.*, 2003; Gomez-Lopez *et al.*, 2010) and in preterm labour (Hamilton *et al.*, 2012). In animal models of preterm birth, decidual macrophage numbers were elevated during labour (Hamilton *et al.*, 2012; Shynlova *et al.*, 2013). Importantly, macrophage depletion protected mice from CPG ODN-induced early pregnancy loss and preterm birth in IL-10 knockout (KO) (Thaxton *et al.*, 2009) and NK-cell deficient non-obese diabetic (NOD) mice (Sun *et al.*, 2013), and LPS-induced preterm delivery was prevented in wild-type mice (Gonzalez *et al.*, 2011).

Uterine natural killer (uNK) cells are another immune cell population that may be involved in the onset of parturition. During early pregnancy, uNK cells are the predominant immune cell population present in the human decidua, with their numbers gradually decreasing towards term (Bulmer *et al.*, 1991; Bartmann *et al.*, 2014). The uNK cells are proposed to play important roles in spiral arteriole remodelling and extravillous trophoblast invasion in early pregnancy (Erlebacher, 2013); however the role of uNKs in late pregnancy and parturition remains unclear. Depletion of uNK cells rescued LPS-induced fetal resorption and preterm birth in IL-10 KO mice (Murphy *et al.*, 2005, 2009), but not CpG ODN-induced fetal resorption or preterm birth in IL-10 KO mice (Thaxton *et al.*, 2009), suggesting that the role of uNK cells may depend on the nature of the inflammatory insult.

Several studies have also investigated the role of cells of the adaptive immune system, T and B cells, in pregnancy and parturition. Both T and B cells are found in the human myometrium, decidua and fetal membranes, with the number of T cells reported to increase in these tissues in association with the onset of labour at term (Thomson *et al.*, 1999; Gomez-Lopez *et al.*, 2013a, b). Regulatory T cells (Tregs), which are defined as CD4^+^CD25^bright^FoxP3^+^ cells, appear to play an important, immunosuppressive role in the maintenance of pregnancy (Erlebacher, 2013), with animal studies demonstrating that depletion of FoxP3^+^ cells results in early pregnancy loss (Aluvihare *et al.*, 2004; Rowe *et al.*, 2012), and adoptive transfer of Tregs rescues CpG-ODN-induced pregnancy loss in NOD mice (Lin *et al.*, 2014). Other subpopulations of T cells, such as Th17 cells (CD3^+^CD4^+^IL-17A^+^), CD4^+^ cells and CD8^+^ cytotoxic T cells have also been identified at the maternal–fetal interface, but their precise role in the maintenance of pregnancy and/or onset of labour at term and preterm, remains unclear (Gomez-Lopez *et al.*, 2014). Interestingly, *Rag1* KO mice, which are deficient in both T and B cells, were not protected from LPS-induced preterm delivery, and were actually found to be more likely (than their wild-type counterparts) to deliver preterm following low-dose LPS (an effect which was partially reversed following adoptive transfer of CD4+ cells) (Bizargity *et al.*, 2009). Further work is needed to elucidate the precise functions of these adaptive immune cell populations during pregnancy and parturition.

Taken together, the above studies highlight the diverse immune cell populations, present at the maternal–fetal interface, which have the potential to be involved in the initiation of parturition. There will likely be differences in the function of each immune cell population in the presence of an intrauterine infection, compared with their role during spontaneous term labour, however the animal studies discussed here have provided promising evidence regarding the role of iNKT cells in the onset of inflammation-induced preterm birth, with some evidence suggesting macrophages and uNK cells may also play a role. For neutrophils, mast cells and T cells, although they have also been implicated in parturition, likely redundancy is demonstrated by studies where their depletion does not alter the timing of preterm parturition.

Whilst preterm birth remains the leading cause of neonatal mortality and morbidity worldwide, there is an urgent need for novel treatments (Blencowe *et al.*, 2013; Norman and Shennan, 2013). Therefore, studies identifying the underlying mechanisms leading to preterm birth, such as the work by Li *et al.*, published in this issue, are critical in highlighting new potential therapeutic targets. Although the precise role of iNKT cells in preterm birth in women remains to be elucidated, improved understanding of the role of individual immune cell populations in the onset of preterm birth may lead to the identification of novel therapeutic targets and ultimately the development of new treatments to delay preterm delivery and improve neonatal outcome.

## Authors’ roles

S.F.R., A.G.R., P.T.K.S. and J.E.N. all contributed to the preparation of the manuscript and gave final approval of the version to be published.

## Funding

S.F.R. is supported by Medical Research Council (grant number MR/L002657/1) and Tommy's the baby charity. Funding to pay the Open Access publication charges for this article was provided by the Medical Research Council.

## Conflict of interest

J.E.N., A.G.R. and P.T.K.S. hold a Medical Research Council grant investigating iNKT cells as drivers for preterm labour. S.F.R. is employed under this grant (Grant number MR/L002657/1).
